# Acetylation of PPARγ in macrophages promotes visceral fat degeneration in obesity

**DOI:** 10.1093/lifemeta/loac032

**Published:** 2022-11-11

**Authors:** Nicole Aaron, Tarik Zahr, Ying He, Lexiang Yu, Brent Mayfield, Utpal B. Pajvani, Li Qiang

**Affiliations:** 1 Naomi Berrie Diabetes Center, Columbia University, New York, NY, USA; 2 Department of Pharmacology, Columbia University, New York, NY, USA; 3 Department of Pathology and Cell Biology, Columbia University, New York, NY, USA; 4 Department of Genetics and Development, Columbia University, New York, NY, USA; 5 Department of Medicine, Columbia University, New York, NY, USA

**Keywords:** PPARγ acetylation, adipose tissue remodeling, macrophage, inflammation, fibrosis

## Abstract

Obesity is characterized by chronic, low-grade inflammation, which is driven by macrophage infiltration of adipose tissue. PPARγ is well established to have an anti-inflammatory function in macrophages, but the mechanism that regulates its function in these cells remains to be fully elucidated. PPARγ undergoes post-translational modifications (PTMs), including acetylation, to mediate ligand responses, including on metabolic functions. Here, we report that PPARγ acetylation in macrophages promotes their infiltration into adipose tissue, exacerbating metabolic dysregulation. We generated a mouse line that expresses a macrophage-specific, constitutive acetylation-mimetic form of PPARγ (*K293Q*^*flox/flox*^*:LysM-cre*, mK293Q) to dissect the role of PPARγ acetylation in macrophages. Upon high-fat diet feeding to stimulate macrophage infiltration into adipose tissue, we assessed the overall metabolic profile and tissue-specific phenotype of the mutant mice, including responses to the PPARγ agonist Rosiglitazone. Macrophage-specific PPARγ K293Q expression promotes proinflammatory macrophage infiltration and fibrosis in epididymal white adipose tissue, but not in subcutaneous or brown adipose tissue, leading to decreased energy expenditure, insulin sensitivity, glucose tolerance, and adipose tissue function. Furthermore, mK293Q mice are resistant to Rosiglitazone-induced improvements in adipose tissue remodeling. Our study reveals that acetylation is a new layer of PPARγ regulation in macrophage activation, and highlights the importance and potential therapeutic implications of such PTMs in regulating metabolism.

## Introduction

Obesity and its associated comorbidities represented by type 2 diabetes mellitus, cardiovascular diseases and some forms of cancer are a public health concern. Obesity, which is caused by an imbalance between caloric intake and energy expenditure, is typically associated with chronic, low-grade inflammation that ultimately drives metabolic dysfunction and insulin resistance [1, 2]. In hypertrophic adipose tissue depots, infiltration of adipose tissue macrophages (ATMs) is considered both a marker and a pathogenic determinant of adipose tissue inflammation [3, 4], leading to exacerbated metabolic impairment. In addition to being the main source of inflammatory cytokines in adipose tissue [5, 6], ATMs participate in the regulation of lipid and glucose metabolism in adipocytes [7, 8]. Despite recent advances in the study of ATMs, the underlying mechanism by which macrophages are recruited into adipose tissues and how they subsequently promote tissue degeneration has not yet been fully elucidated.

The nuclear receptor peroxisome proliferator-activated receptor γ (PPARγ) plays a dominant role in adipose tissue development and remodeling. Highly expressed in adipocytes, it is recognized as a master regulator of adipocyte differentiation and function, including adipokine production and lipid and glucose metabolism [9, 10]. In addition to adipocytes, PPARγ is also relatively well-expressed in macrophages [11–13]. Pharmacological activation of PPARγ by thiazolidinediones (TZDs), an important class of anti-diabetic drugs, has been shown to attenuate the inflammatory response of macrophages [11, 13]. *In vivo*, TZD treatment improved adipose tissue response to high-fat diet (HFD) feeding and promoted alternative activation of macrophages [14], likely mediated by PPARγ agonism in macrophages [15]. Congruently, the deletion of PPARγ in macrophages has been shown to impair lipid catabolic metabolism, leading to metabolic dysfunction in response to HFD feeding [16]. In human studies, PPARγ activation induces a macrophage response characteristic of alternative (M2-like) activation [17]. Thus, PPARγ is a critical regulator in the anti-inflammatory response of macrophages, ultimately dictating the overall metabolic health of adipose tissue.

The regulation of PPARγ activity in macrophages is primarily studied at the level of ligand-dependent agonism, despite a complex regulatory machinery. For example, we previously identified two residues on PPARγ, Lys268, and Lys293, that are deacetylated by the NAD^+^-dependent deacetylase, SirT1 [18]. Constitutive PPARγ deacetylation (K268R/K293R, 2KR) improves the metabolic phenotype of a mouse model of diet-induced obesity (DIO) [19]. PPARγ acetylation, on the other hand, is commonly observed in obesity, diabetes, and aging [18], all of which are conditions accompanied by macrophage activation. Thus, we hypothesized that these post-translational modifications (PTMs) of PPARγ in macrophages are involved in regulating adipose tissue health. Here, we generated mice expressing a macrophage-specific, constitutive acetylation-mimetic mutant form of PPARγ to systemically investigate, *in vivo* and *in vitro,* the function of PPARγ acetylation in macrophages. By utilizing this model, we show that macrophage PPARγ Lys293 acetylation is a causal factor involved in deteriorating adipose tissue integrity during obesity. Together, these findings reveal that PPARγ acetylation is a novel regulator of macrophage activation, ultimately influencing adipose tissue function.

## Results

### Macrophage-specific PPARγ acetylation impairs energy expenditure in response to HFD feeding

**Figure 1:**
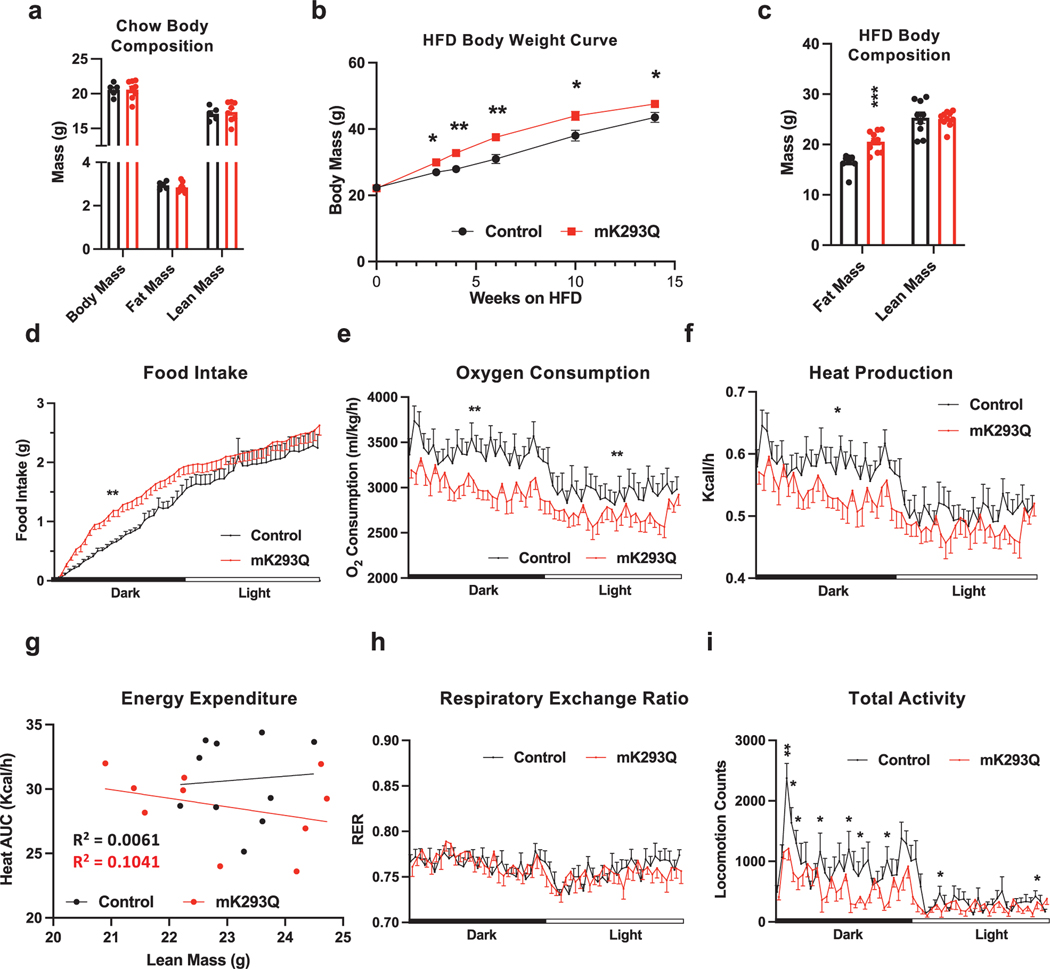
mK293Q mice have an impaired metabolic response to HFD feeding. (a) BW and composition assessed by EchoMRI of 28-week-old male mice fed a chow diet *ad libitum*, control (*n* = 5), mK293Q (*n* = 8). (b and c) Male mice were fed a HFD for 16 weeks, control (*n* = 9), mK293Q (*n* =10). BW curve (b); body composition assessed by EchoMRI (c). (d−h) Calorimetric analyses of adult male mice at ambient temperature on an 8-week HFD, control (*n* =10), mK293Q (*n* = 10). All measurements were taken from the same light/dark cycle. Food intake (d); oxygen consumption (e); heat production (f) and the area under the curve (AUC) distribution to lean mass (g). Simple linear regression analysis was computed with a 95% confidence interval, (Black = control, Red = mK293Q). (h) RER; (i) Total activity. *P < 0.05, **P < 0.01 for control group vs mK293Q mice. Data represent mean ± SEM. Two-tailed Student’s *t*-tests were used for statistical analyses.

Given the important function of PPARγ deacetylation in promoting catabolism in adipocytes and the crucial role that PPARγ plays in macrophages, we hypothesized that PPARγ acetylation is involved in regulating the activity of ATMs. To directly test this hypothesis, we generated knock-in mice that express a macrophage-specific, constitutive acetylation-mimetic mutant form of PPARγ by breeding mice carrying a floxed allele of the K293Q mutation with the *LysM-cre* line to target myeloid-derived cells, resulting in *K293Q*^*flox/flox*^*;LysM-cre* (mK293Q) mice (Supplementary Fig. S1a). We confirmed the replacement of wild-type (WT) PPARγ by mutant K293Q in bone marrow-derived macrophages (BMDMs) (Supplementary Fig. S1b). We targeted the lysine residue of PPARγ at position 293 given its known role as one of the most responsive acetylation sites involved in dictating PPARγ function [18]. Normal chow-fed mK293Q mice showed no difference in body weight (BW) and composition compared to normal chow-fed control *K293Q*^*flox/flox*^ mice (Fig. 1a). As lean mice show low basal macrophage infiltration into adipose tissue and little macrophage activation, we challenged mK293Q mice and control *K293Q*^*flox/flox*^ mice with HFD feeding. HFD feeding-induced weight gain was exacerbated in mK293Q mice at all time points measured during the feeding regimen, which was due specifically to a higher fat mass (Fig. 1b and c).

Previous research has drawn an association between ATMs and metabolic activity [20]. To understand the phenotype in HFD-fed mK293Q mice, we performed indirect calorimetry. Interestingly, although total daily food intake remained the same, the eating pattern was altered in mK293Q mice, with more food intake in the dark phase (Fig. 1d). We also noted a modest impairment of oxygen consumption and heat production in mK293Q mice (Fig. 1e-g), without affecting the respiratory exchange ratio (RER) (Fig. 1h). Interestingly, the total locomotor activity of mK293Q mice on a HFD was significantly dampened (Fig. 1i). Overall, these data demonstrate that constitutive macrophage PPARγ acetylation reduces energy expenditure and exacerbates weight gain and fat mass accrual during HFD feeding.

### Macrophage infiltration into the eWAT is greater in mK293Q mice

**Figure 2:**
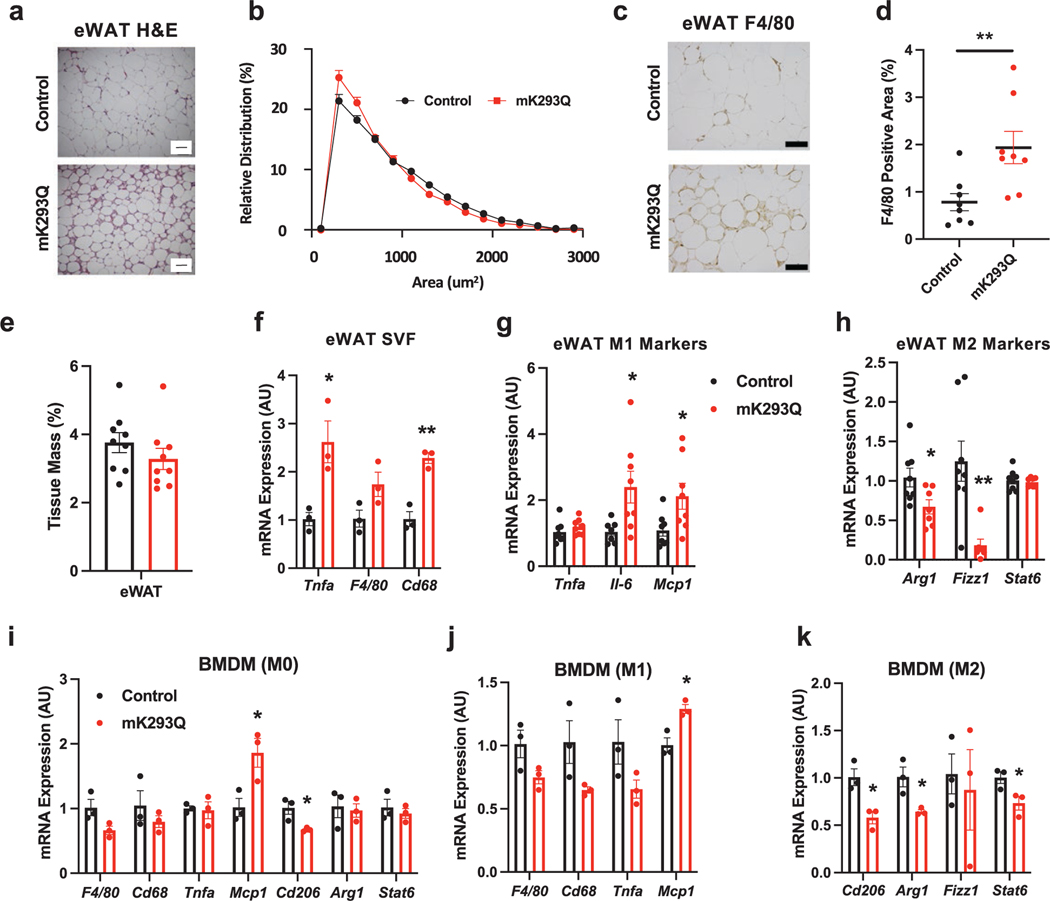
mK293Q promotes macrophage infiltration and inflammation in eWAT upon HFD feeding. (a−d) Male mice were fed a HFD for 16 weeks. Representative images (a) and adipocyte size quantification (b) from H&E-stained eWAT (*n* = 8, 8). Immunohistochemistry staining of F4/80 in eWAT (c) and quantification (d) (*n* = 8, 8). quantification of eWAT depot size normalized to BW (*n* = 9, 9, one mouse in the mK293Q mice was missed to measure) (d). (f) qPCR analyses of macrophage-related gene expression from SVF cells isolated from the eWAT of male mice fed a HFD for 8 weeks (*n* = 3, 3). (g and h) qPCR analyses of gene expression for markers of proinflammatory (g) and anti-inflammatory (h) macrophage function in the eWAT of mice on a HFD for 16 weeks (*n* = 8, 8). (i−k) qPCR analyses of macrophage-related gene expression from non-activated (M0) BMDMs (i), proinflammatory M1 activated macrophages (j), and anti-inflammatory M2 activated macrophages (k) (*n* = 3, 3). *P < 0.05, ** *P* < 0.01 for control group vs mK293Q mice. Scale bars = 200 μm in length. Data represent mean ± SEM. Two-tailed Student’s *t*-tests were used for statistical analyses.

We focused our initial analysis on epididymal white adipose tissue (eWAT), as visceral fat is more prone to macrophage infiltration and detrimental to metabolic health [21–23]. Based on the phenotype of mK293Q mice, we initially predicted these mice would display adipocyte hypertrophy, but Harris hematoxylin and eosin (H&E) staining of the eWAT showed a smaller cell size in mK293Q mice (Fig. 2a and b). Adipocytes from mK293Q mice also appeared atrophic, with prevalent “crown-like” structures—a key feature of macrophage infiltration—accompanied by greater immunostaining for the macrophage marker F4/80 (Fig. 2c and d). There was, however, no observed difference in overall eWAT size when normalized to BW (Fig. 2e). Consistent with this observation, isolated cells from the adipose stromal vascular fraction (SVF) of the eWAT from mK293Q mice, which contain non-adipocytes, including macrophages, displayed greater expression of the proinflammatory gene *Tnfa* and the macrophage markers *F4/80* and *Cd68* (Fig. 2f). These data suggest that PPARγ activity is dysfunctional in mK293Q mice, as PPARγ activation is thought to suppress inflammation by lowering the number of ATMs [4, 24].

PPARγ is known to favor the alternative polarization of macrophages [17]. Further analysis in mK293Q mice revealed increased expression of the M1-associated proinflammatory genes *Il-6* and *Mcp1* (Fig. 2g), as well as decreased expression of the anti-inflammatory M2 polarization genes *Arg1* and *Fizz1* (Fig. 2h). We, therefore, hypothesized that PPARγ acetylation directly impedes this alternative activation in macrophages. BMDMs from mK293Q mice displayed little differences in the basal state (Fig. 2i)—aside from significantly increased expression of *Mcp1*, which encodes for Monocyte Chemotactic Protein 1 (MCP-1), known to be involved in macrophage recruitment [20, 25–27]. Similarly, M1 activation by lipopolysaccharides (LPS) stimulation did not result in a marked gene expression difference in mK293Q BMDMs except for the consistent upregulation of *Mcp1* (Fig. 2j). In contrast, treatment of mK293Q BMDMs with interleukin-4 (IL-4) to drive M2 polarization resulted in impaired expression of the anti-inflammatory genes *Cd206*, *Arg1*, and *Stat6* (Fig. 2k).

Dysfunctional mitochondrial activity, including oxidative phosphorylation, contributes to a dysregulated inflammatory response in macrophages and can promote an imbalanced bias towards an M1-like gene program [28–31]. Interestingly, the expression of *Tomm20*, *Atp5a1*, and *Cox5b*, three key genes involved in mitochondrial activity, remained mostly unaffected in mK293Q BMDMs compared to control BMDMs from WT mice (Supplementary Fig. S2). Together, these data suggest that PPARγ acetylation promotes macrophage infiltration and proinflammatory activation in eWAT.

### eWAT function is impaired in mK293Q mice

**Figure 3:**
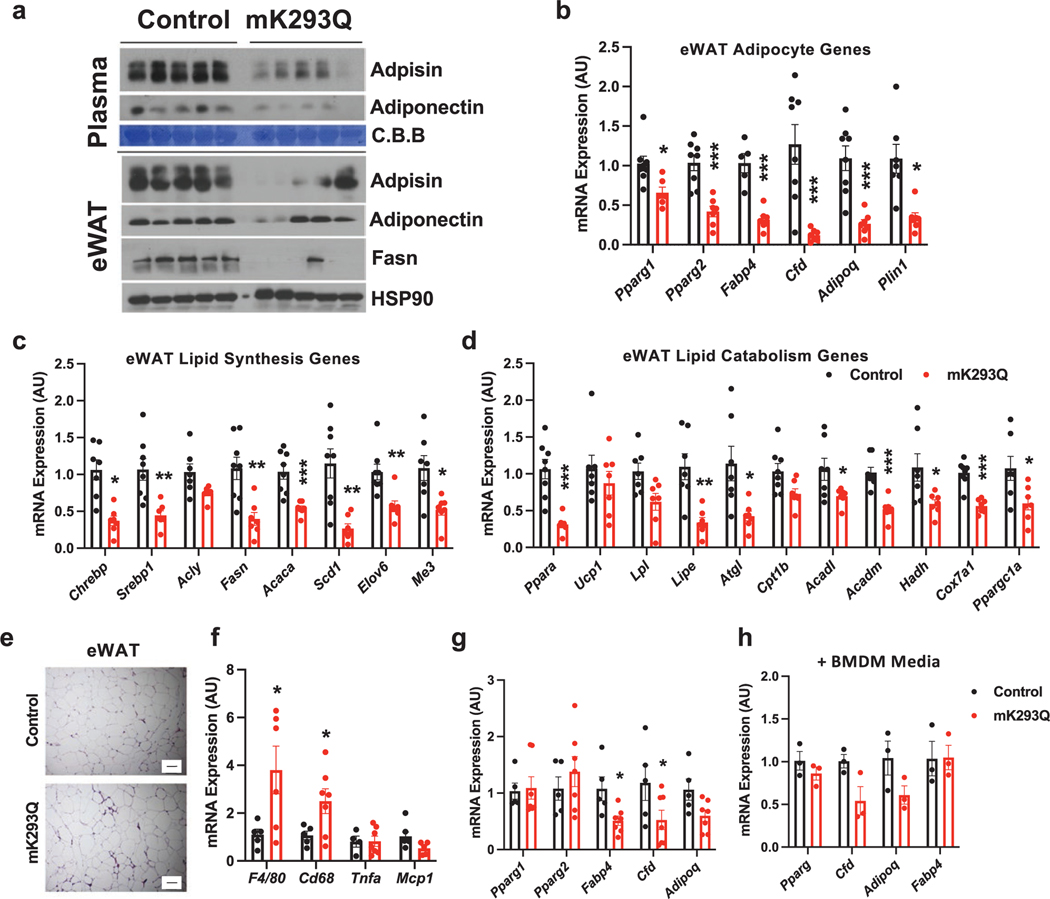
PPARγ acetylation in macrophages impairs adipose tissue function.(a−d) Male mice were placed on 16-week HFD feeding. Immunoblot of the adipokines Adipsin and Adiponectin in the plasma and Adipsin, Adiponectin and Fasn in the eWAT. Coomassie Brilliant Blue (C.B.B, plasma) and HSP90 (eWAT) were used as the loading controls (a). qPCR analyses of expression for adipogenic markers in eWAT (b). qPCR analyses of gene expression for markers of lipid synthesis (c) and oxidation (d) in eWAT (*n* = 8, 8). (e−g) representative images of H&E-stained eWAT (e). qPCR analyses of expression of inflammatory markers (f) and adipocyte genes (g) in eWAT (*n* = 5, 7) in 8-week HFD-fed mice. (h) qPCR analyses of expression of adipogenic markers in adipogenic 3T3-L1 with conditioned media from Control and K293Q BMDMs (*n* = 3, 3). Scale bars = 200 μm in length. *P < 0.05, ** *P* < 0.01, *** *P* < 0.001 for control group vs mK293Q mice. Data represent mean ± SEM. Two-tailed Student’s *t*-tests were used for statistical analyses.

Increased macrophage infiltration leads to impaired adipose function [3, 4]. As expected, the production of two key adipokines that regulate systemic insulin sensitivity and glucose homeostasis [32], Adiponectin (encoded by *Adipoq*) and Adipsin (encoded by *Cfd*), was decreased in the plasma of mK293Q mice compared to control mice and a strong reduction in Adipsin and Fasn (the latter being involved in fatty acid synthesis) was observed in the eWAT of mK293Q mice compared to controls, while Adiponectin levels were only mildly affected in the mutant mice (Fig. 3a). In addition, we observed a broad repression of adipogenic gene expression, including in *Pparg1, Pparg2, Fabp4, Cfd, Adipoq*, and *Plin1* (Fig. 3b). A signature function of adipose tissue is storage and release of lipids. Interestingly, most of the genes involved in fatty acid synthesis (*Chrebp*, *Srebp1*, *Fasn*, *Acaca*), desaturation (*Scd1*) and, elongation (*Elov6*) were repressed in the eWAT of mK293Q mice (Fig. 3c). In parallel, genes associated with lipid release (*Atgl*, *Lipe*), oxidation (*Ppara*, *Acadl*, *Acadm*, *Hadh*), and mitochondrial activity (*Ppargc1a*, *Cox7a1*) were also downregulated (Fig. 3d), indicating impaired lipid metabolism overall. These results highlight the role of PPARγ acetylation in macrophages to drive adipose tissue degeneration, consistent with established roles of macrophage PPARγ in whole-body lipid metabolism [16].

To understand whether the adipocyte phenotype of mK293Q mice is secondary to the development of obesity, we assessed the eWAT phenotype of control and mK293Q mice fed a HFD for only 8 weeks. At this time point, we observed no change in adipocyte size, but still a slight increase in the extent of crown-like structures by H&E staining (Fig. 3e), indicative of accelerated macrophage infiltration into the eWAT of mK293Q mice. Consistent with these results, the gene expression of the macrophage markers *F4/80* and *Cd68* was higher in the eWAT of mK293Q mice compared to control mice (Fig. 3f), but adipogenic markers were less affected than that of 16-week HFD-fed mice (Fig. 3g, compared to Fig. 3b). Furthermore, treatment of differentiating 3T3-L1 pre-adipocytes with conditioned media collected from non-polarized, differentiated control and K293Q BMDMs confirmed the absence of significant adipogenic changes *in vitro* (Fig. 3h). Given these milder effects observed during the early-stages of inflammation and the absence of phenotypical differences on chow diet, we conclude that the dysfunction of eWAT caused by PPARγ acetylation in macrophages is dependent on macrophage infiltration, which is exaggerated by long-term HFD feeding.

### Adipose tissue fibrosis is exacerbated in DIO mK293Q mice

**Figure 4:**
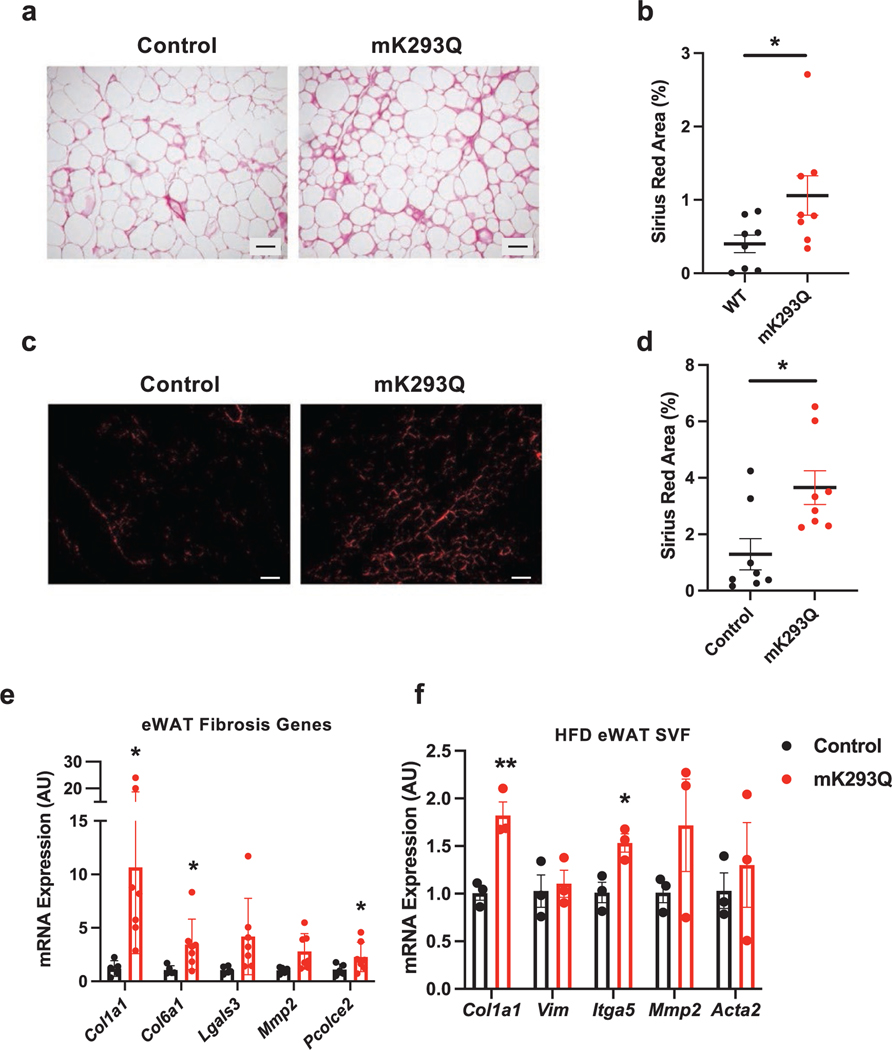
mK293Q mice have elevated fibrosis in the eWAT. (a−d) In 16-week HFD-fed mice, representative images from picrosirius red staining of eWAT imaged by brightfield (a), quantified (*n* = 8, 8) (b) and imaged by polarized-light microscopy (c), quantified (*n* = 8, 8) (d). (e and f) qPCR analyses of genes associated with fibrosis in eWAT (*n* = 5, 7) (e) and SVFs (*n* = 3, 3) (f) after 8 weeks on HFD feeding. Scale bars = 200 μm in length. *P < 0.05, **P < 0.01 for control group vs mK293Q mice. Data represent mean ± SEM. Two-tailed Student’s *t*-tests were used for statistical analyses.

Adipose tissue inflammation leads to fibrosis, which drives further dysregulation [33, 34], including insulin resistance [35]. The morphology of the eWAT of mK293Q mice after 16-week HFD feeding showed enlarged intercellular mesenchymal space (Fig. 2a), raising the possibility of increased fibrosis. Picrosirius red staining revealed increased collagen deposition in the eWAT of mK293Q mice (Fig. 4a and b). By polarized microscopy, we imaged and quantified the fibrotic area to reveal an over 2-fold increase in the mutant mice compared to the WT mice (Fig. 4c and d). Notably, this accelerated fibrogenic process started as early as 8 weeks on HFD feeding, indicated by a striking 10-fold induction of *Col1a1* and other important fibrogenic genes, including *Col6a1* and *Pcolce2* (Fig. 4e). Further, cells from the SVF isolated from the eWAT of 8-week HFD-fed mice showed a consistently greater expression of fibrogenic genes in mK293Q mice compared to controls (Fig. 4f). It should be noted that there were no observed differences in the adipogenic capacity of adipocyte progenitor cells isolated from the mutant mice and controls on either HFD or chow feeding (data not shown). Thus, increased inflammation in mK293Q mice drives the development of adipose tissue fibrosis that exacerbates the impairment of adipose function, most likely by affecting its plasticity and associated metabolic response.

### PPARγ acetylation in macrophages increases obesity-associated hepatic steatosis

**Figure 5:**
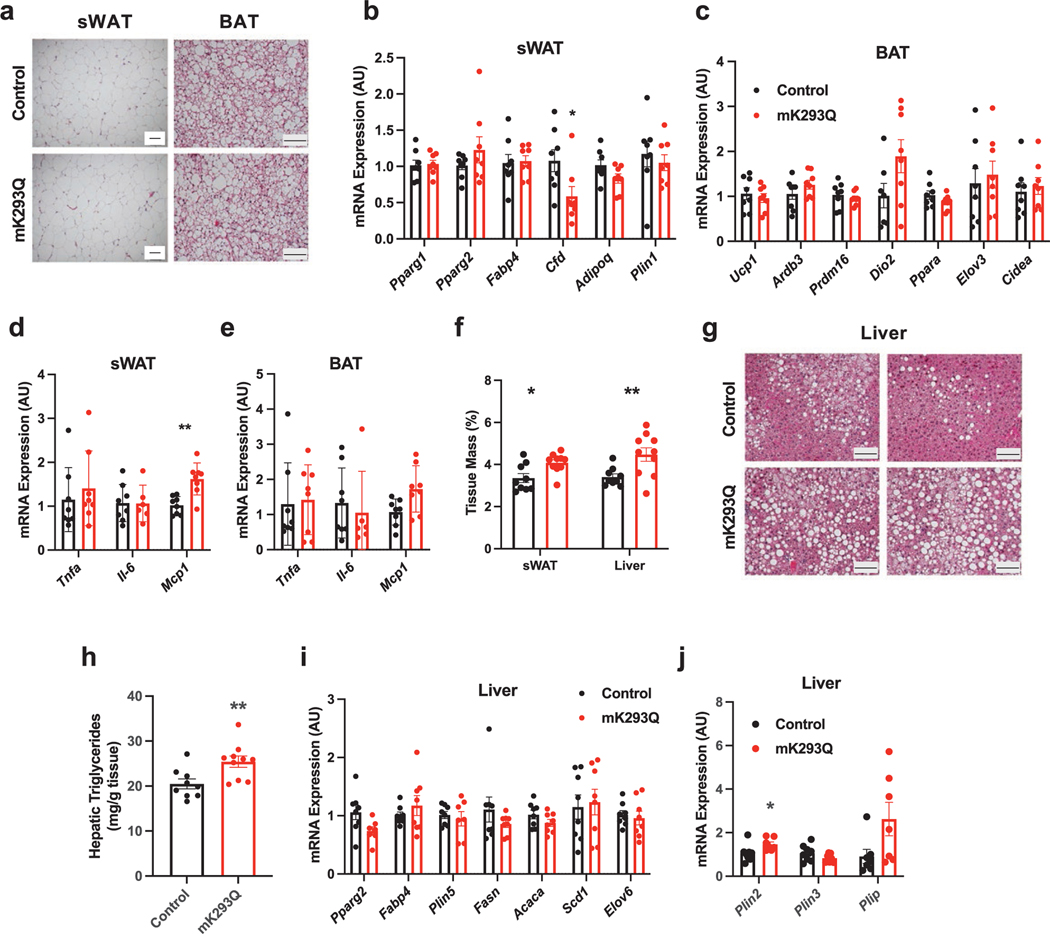
mK293Q mice develop aggravated hepatic steatosis with HFD feeding. Male mice were fed a HFD for 16 weeks. (a) Representative images from H&E-stained subcutaneous inguinal white adipose tissue (sWAT) and BAT. (b−e) qPCR analyses of expression of adipogenic genes in the sWAT (b), thermogenic genes in the BAT (c) and genes involved in inflammation in both tissue types (d) and (e) (*n* = 8, 8). (f) quantification of sWAT depot size and liver mass normalized to total BW (*n* = 9, 10). (g and h) Representative images from H&E-stained liver tissue (g) and quantification of hepatic triglyceride content (h) (*n* = 9, 10). (i and j) qPCR analyses of genes involved in (i) hepatic steatosis and (j) lipid droplet formation (*n* = 8, 8). Scale bars = 200 μm in length. *P < 0.05, **P < 0.01 for control group vs mK293Q mice. Data represent mean ± SEM. Two-tailed Student’s *t*-tests were used for statistical analyses.

HFD feeding induces much less macrophage infiltration in subcutaneous white adipose tissue (sWAT) and brown adipose tissue (BAT) than in eWAT [21]. We observed minimal morphological changes in the sWAT and BAT of mK293Q mice despite prolonged 16-week HFD feeding (Fig. 5a). The expression of adipogenic genes in sWAT (Fig. 5b) and thermogenic genes in BAT (Fig. 5c) remained mostly unchanged in mK293Q mice. In addition, inflammation remained mostly unaffected in both sWAT and BAT, except for a persistent upregulation of *Mcp1* expression in the sWAT (Fig. 5d and e), in line with the increased sWAT depot size (Fig. 5f). This further elucidates a connection between PPARγ acetylation and macrophage infiltration into adipose tissue, likely through *Mcp1*. However, the absence of metabolic gene expression changes in the sWAT and BAT confirms that the metabolic dysfunctions in mK293Q mice likely arise from eWAT degeneration.

In contrast to the sWAT and BAT, the liver of the mutant mice showed increased tissue size and worsened hepatic steatosis in HFD-fed mK293Q mice compared to controls (Fig. 5f-h). Interestingly, however, there were no changes in genes associated with lipid synthesis in the refed state (Fig. 5i), suggesting that the steatosis phenotype was not caused by increased hepatic *de novo* lipogenesis, but rather due to impaired lipid storage capacity in eWAT of mK293Q mice, driving excess fat accumulation in the liver. Moreover, the expression of *Plin2*, which encodes the major lipid droplet-binding protein Adipocyte Differentiation Related Protein in hepatocytes [36], was increased in the liver of DIO mK293Q mice (Fig. 5j), affirming the hepatic steatosis phenotype. These results not only highlight eWAT as the major adipose tissue affected by PPARγ acetylation in macrophages, but also provide a prospective crosstalk between PPARγ acetylation and hepatic health.

### Impaired metabolic responses in DIO mK293Q mice

**Figure 6:**
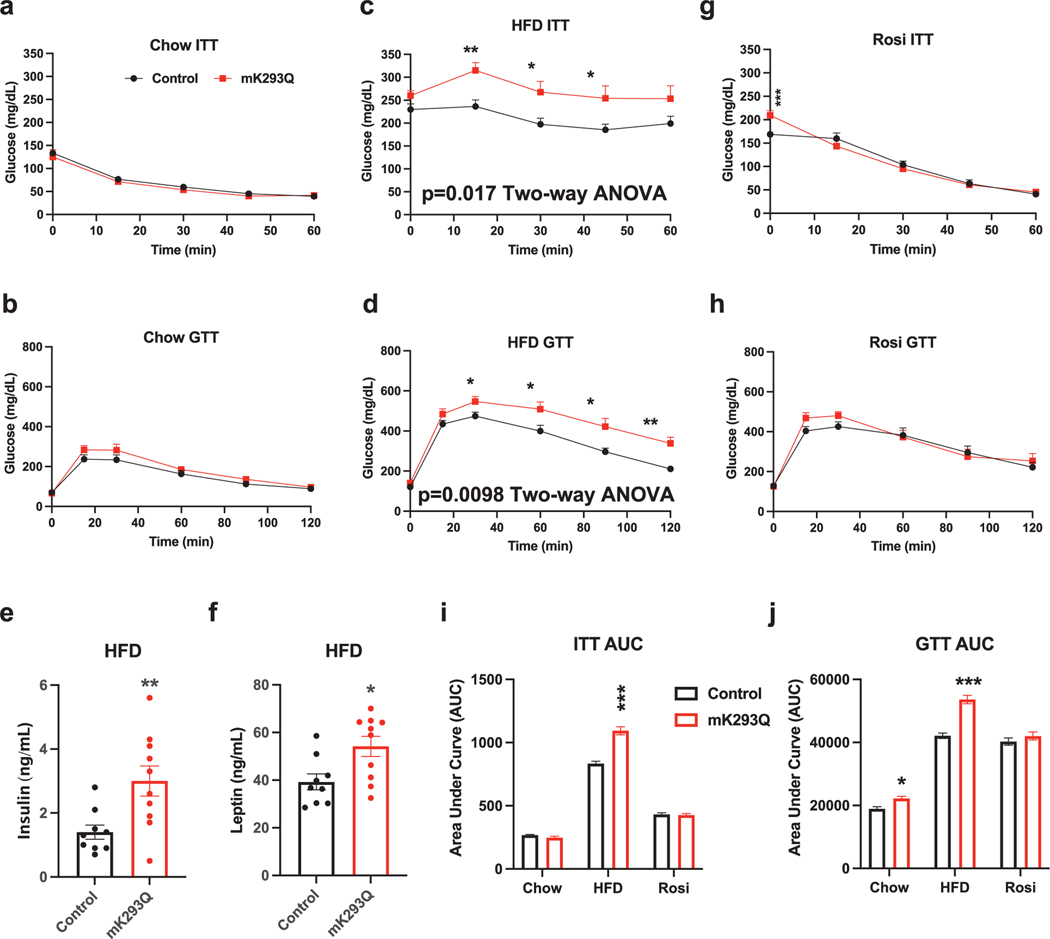
Prevention of insulin resistance and glucose intolerance in DIO mK293Q mice with TZD treatment. (a and b) ITT (a) and GTT (b) in 28-week-old male mice fed a chow diet *ad libitum*, control (*n* = 5), mK293Q (*n* = 8). Two-way ANOVA with *P*-value for column factor not significant. (c and d) ITT (c) and GTT (d) of 16-week HFD-fed male mice, control (*n* = 9), mK293Q (*n* = 10). Two-way ANOVA with *P*-value for column factor = 0.017 (ITT) and = 0.0098 (GTT). (e and f) Plasma insulin (e) and Leptin measurements (f) in HFD-fed control and mK293Q mice (*n* = 9, 10). (g and h) ITT (g) and GTT (h) of male mice rendered obese by HFD feeding for 8 weeks followed by 4 weeks of HFD supplemented with Rosiglitazone (Rosi), control (*n* = 10), mK293Q (*n* = 10). Two-way ANOVA with *P*-value for column factor not significant. (i and j) Quantified Area Under the Curve (AUC) of all ITTs (i) and GTTs (j). *P < 0.05, **P < 0.01, ***P < 0.001 for control group vs mK293Q mice. Data represent mean ± SEM. Two-tailed Student’s *t*-tests and Two-way ANOVA tests were used for statistical analyses.

Chow-fed mK293Q mice showed normal insulin and glucose tolerance (Fig. 6a and b), which is unsurprising given the normal BW distribution (Fig. 1a) and lower macrophage infiltration of basal conditions [3]. But with long-term HFD feeding, both insulin sensitivity and glucose tolerance in mK293Q mice were impaired (Fig. 6c and d). The worsened insulin resistance in HFD-fed mK293Q mice was further evidenced by an increase in plasma insulin levels (Fig. 6e). Additionally, plasma leptin levels were significantly increased in mK293Q mice, in line with their increased adiposity [37] (Fig. 6f). Given the involvement of macrophages in adipose tissue biology and our previous observation that acetylation on the Lys293 residue is a direct target of PPARγ TZD agonist [18], we hypothesized that constitutive PPARγ acetylation would blunt the glucose-lowering effects of TZD. However, treatment of mK293Q mice with the TZD Rosiglitazone (Rosi) for 4 weeks was sufficient to normalize their impaired insulin sensitivity and glucose tolerance (Fig. 6g-j). Ultimately, these results suggest that systemic application of a potent PPARγ agonist (namely, TZD) overrides the metabolic effects of insulin resistance and glucose intolerance of PPARγ acetylation in macrophages.

### Acetylation of PPARγ in macrophages impairs eWAT responses to TZD treatment

**Figure 7:**
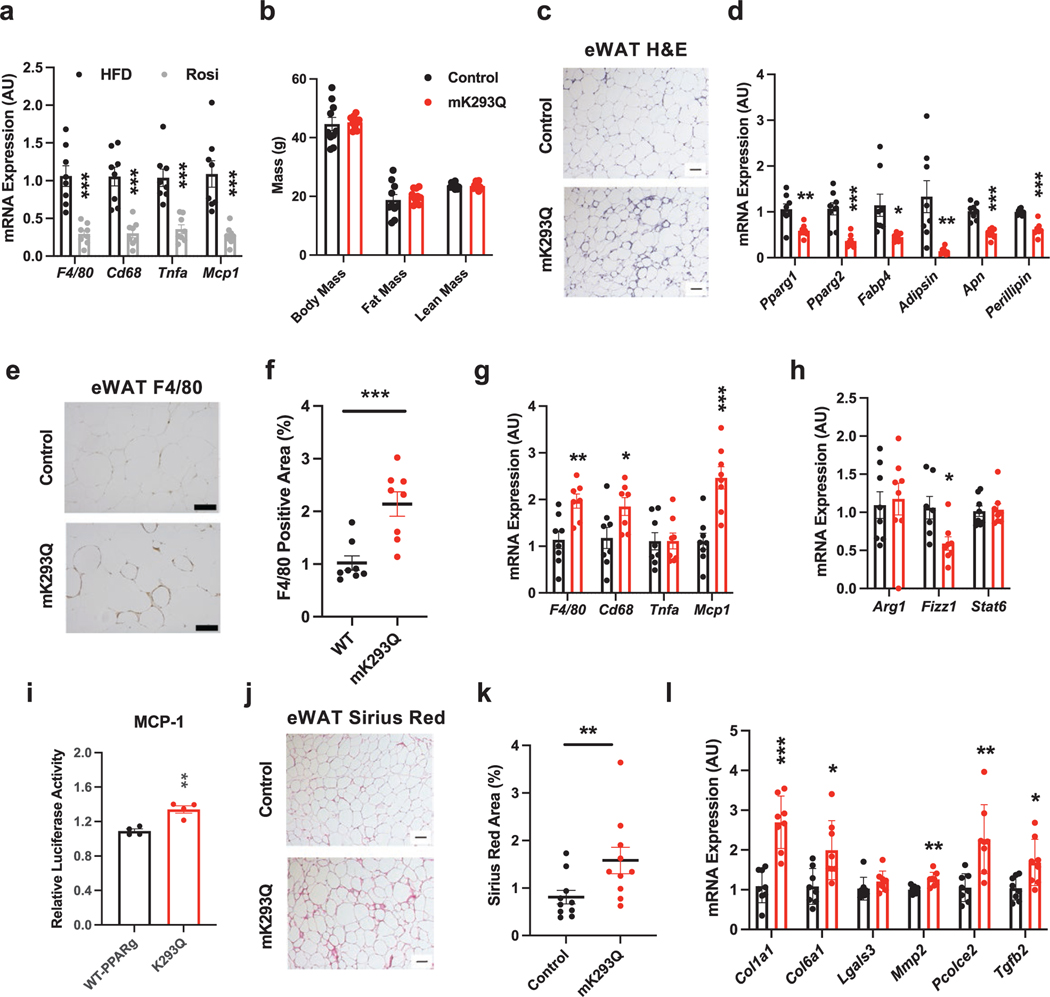
mK293Q mice are less responsive to TZD-induced rescue of adipose tissue health. (a) qPCR analyses of gene expression for markers of inflammation in eWAT from HFD control (*n* = 8) and Rosi-treated (*n* = 8) mice. (b−k) Male mice were rendered obese by HFD feeding for 8 weeks followed by 4 weeks of HFD supplemented with Rosiglitazone (Rosi). (b) BW and composition assessed by EchoMRI (*n* = 10, 10); (c) representative images from H&E-stained eWAT; (d) qPCR analyses of expression for adipocyte genes in eWAT (*n* = 8, 8); (e and f) immunohistochemistry staining of F4/80 in eWAT (e) and quantification (f) (*n* = 8, 8). (g and h) qPCR analyses of gene expression for markers of proinflammatory (g) and anti-inflammatory (h) macrophage functions (*n* = 8, 8). (i) *Mcp-1* promoter luciferase reporter assay in HEK293T cells transfected with WT PPARγ or K293Q mutant (*n* = 4, 4). (j and k) Representative images from picrosirius red-stained eWAT (j) and quantification (k) (*n* = 10, 10). (l) qPCR analyses of genes associated with fibrosis in eWAT (*n* = 8, 8). Scale bars = 200 μm in length. *P < 0.05, **P < 0.01, ***P < 0.001 for control group vs mK293Q mice. Data represent mean ± SEM. Two-tailed Student’s *t*-tests were used for statistical analyses.

TZD treatment can repress the inflammatory response of macrophages and alleviate adipose tissue inflammation [11, 13, 14]. Consistent with these observations, we found that Rosi treatment of HFD-fed WT mice reduced the expression of macrophage markers and inflammatory genes in the eWAT (Fig. 7a). Intriguingly, despite the similar fat mass of HFD-fed control and mK293Q mice in the presence of Rosi (Fig. 7b), we again noted a pronounced increase of “crown-like” structures in the eWAT of mK293Q mice (Fig. 7c) and a parallel repression of adipocyte gene expression (Fig. 7d). Consistent with these data, we observed increased infiltration of macrophages in the eWAT of Rosi-treated mK293Q mice as indicated by F/80 staining (Fig. 7e and f). Further, macrophage markers *F4/80*, *Cd68*, and *Mcp1* were more highly expressed, whereas only the anti-inflammatory marker *Fizz1*, but not *Arg1* or *Stat6*, was less expressed, in the eWAT from Rosi-treated mutant mice compared to controls (Fig. 7g and h). Interestingly, given the consistent upregulation of *Mcp1* in HFD-fed and TZD-treated mK293Q mice, as well as in BMDM cultures, we found an increased activation of the *Mcp1* promoter via PPARγ K293Q in an *in vitro* luciferase reporter assay (Fig. 7i). This finding suggests a direct influence of PPARγ acetylation on *Mcp1* activity, underpinning the exaggerated increase in macrophage infiltration and elevated inflammatory phenotype observed in eWAT of these mice. Furthermore, while Rosi has been demonstrated to have an antifibrotic effect through inhibition of TGF-β signaling [38], eWAT from mK293Q mice treated with Rosi still displayed worsened fibrosis than control mice (Fig. 7j and k), consistent with increased fibrogenic gene expression (Fig. 7l). These results reinforce the inability of Rosi to fully resolve adipose inflammation and fibrosis imposed by PPARγ acetylation, diverging from the normalization of insulin sensitivity and glucose tolerance.

## Discussion

**Figure 8:**
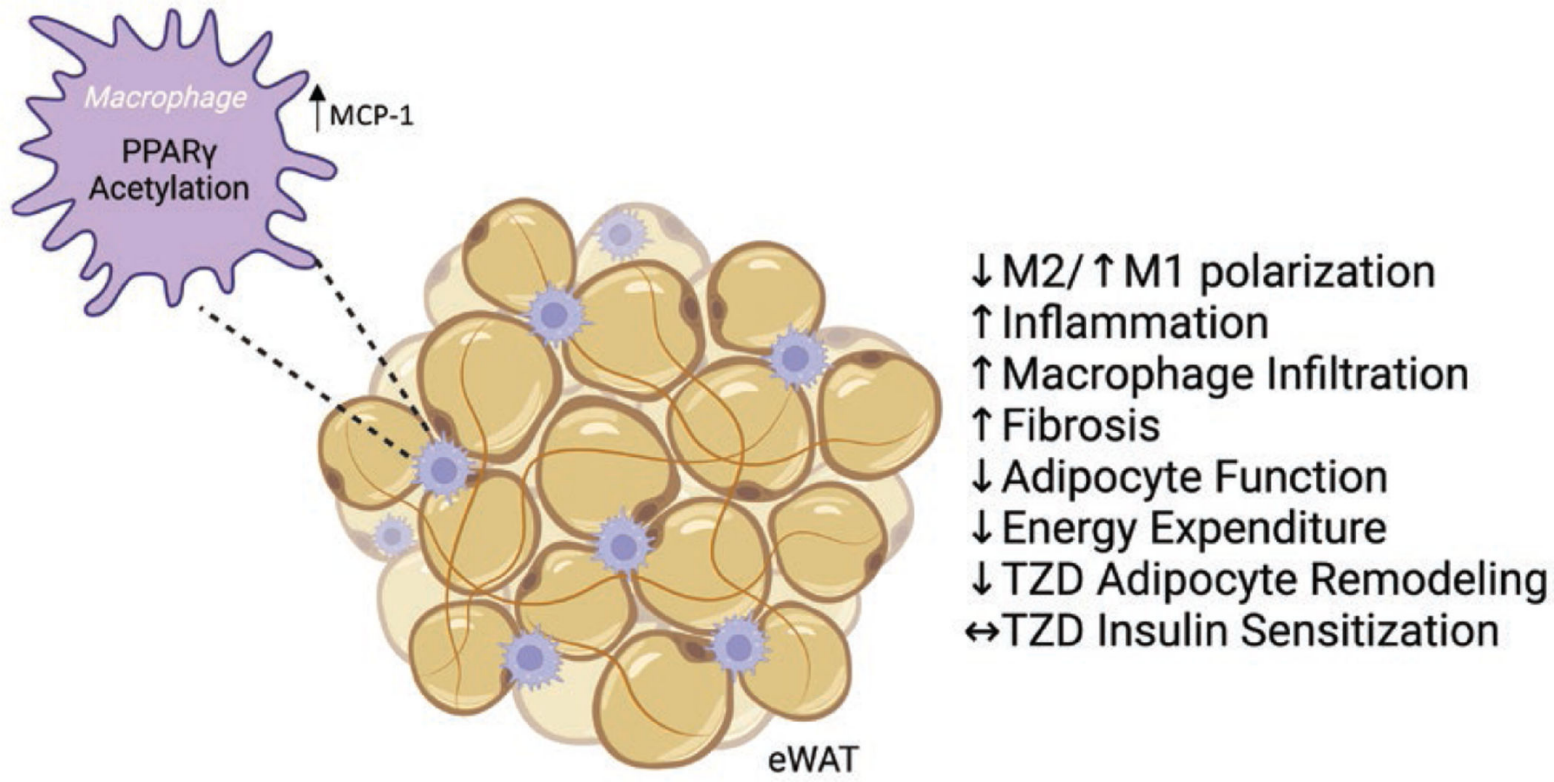
Schematic model. Constitutive acetylation of PPARγ in macrophages promotes M1-like macrophage infiltration in eWAT depots via upregulation of MCP-1 and inhibits M2 polarization during DIO. This condition is associated with increased inflammation and fibrosis, and impaired adipose and metabolic functions, but with continued beneficial responses to TZD-mediated insulin sensitization despite reduced responses to their effect on adipose tissue remodeling.

In this study, we demonstrate the novel role of PPARγ acetylation in macrophages to determine adipose tissue responses. Genetically modified mK293Q mice expressing a constitutive acetylation-mimetic PPARγ mutant in macrophages have an exacerbated response to HFD feeding, with increased BW gain likely due to reduced energy expenditure, and impaired glucose and lipid homeostasis. These metabolic detriments can be traced back to a degeneration of eWAT, likely caused by an increase in macrophage infiltration that leads to worsening of adipose inflammation and fibrosis; further, PPARγ acetylation impedes the ability of macrophages to skew towards an anti-inflammatory phenotype (Fig. 8).

Alternatively activated macrophages are important in maintaining lipid oxidative capacity [39]. Furthermore, we know that the absence of PPARγ in macrophages impairs lipid metabolism entirely [16]. Given the catabolic function of PPARγ deacetylation in adipocytes as demonstrated in our previous studies [18, 19], it is conceivable that PPARγ acetylation is inhibitory of lipid synthesis and oxidation in macrophages, contributing to proinflammatory activation of macrophages in mK293Q mice. Acetylation may deprive the trans-repression of PPARγ on inflammatory genes, in contrast to PPARγ SUMOylation [40]. These mechanisms may act separately or synergistically—the indirect suppression of lipid catabolism or the direct regulation through modification of inflammatory gene expression. Moreover, both acetylation and SUMOylation take place on lysine residues and, thus, may compete for the same residue to dynamically switch PPARγ activity in macrophages. Our work reinforces the importance of PTMs of PPARγ in determining the degree of macrophage activation and metabolic dysregulation and suggests the need for comprehensive studies of PPARγ PTMs in macrophages to further uncover the dynamic regulation of macrophage activity by PPARγ.

Repression of adipocyte genes in the eWAT of mK293Q mice in response to macrophage infiltration became prominent after prolonged HFD feeding, suggesting a novel mechanism of cross-talk between macrophages and adipocytes. One potential mechanism at play is inflammatory cytokine signaling, known to impair adipocyte activity, and consequently, metabolic function. Nevertheless, we noted decreased Adiponectin levels in eWAT of mK293Q mice. Adiponectin has anti-inflammatory actions, promoting M2 polarization [41, 42] and the inhibition of the release of inflammatory cytokines [43]. Furthermore, while Adiponectin and Adipsin levels positively respond to an initial increase in caloric intake, long-term HFD feeding reduces the expression of both [44, 45]. As Adiponectin and Adipsin are decreased in mK293Q mice, these results are suggestive of accelerated response to HFD feeding. It is conceivable that PPARγ acetylation in macrophages may partially explain the paradoxical decrease of adipocyte markers during long-term HFD feeding through cross-talking to adipocytes. Interestingly, treatment with a TZD, Rosiglitazone, did not fully rectify adipose tissue integrity in mK293Q mice, despite improvements in whole-body insulin sensitivity. Regulation of adipocyte fate is primarily dictated by PPARγ [46]. In one report, loss of PPARγ in mice blunted the tissue-specific effects of TZD treatment [47]. Our study suggests that not only the loss of PPARγ in adipocytes but also its acetylation in macrophage can curb the effects of TZDs on remodeling adipose tissue, whereas this adipose tissue remodeling can be uncoupled from the improved whole-body insulin sensitivity.

One of the most interesting observations of our mK293Q mouse model is the striking fibrosis in eWAT in parallel with macrophage infiltration. sWAT does not show this phenotype, likely due to fewer infiltrated macrophages. This result suggests that macrophages drive adipose tissue fibrosis, but additional data suggest a feed-forward loop. TGF-β is a major fibrotic cytokine and has been shown to be released by adipocytes in the proinflammatory state [48]. *Tgfb2* was upregulated in the eWAT of mK293Q mice, although the cellular source of increased *Tgfb2* in eWAT warrants further investigation. PPARγ promotes adipose tissue-derived stromal cell differentiation toward adipocytes at the expense of fibroblasts [49], but we did not detect an impairment in their adipogenic capacity. In primary adipocyte studies, adipocyte progenitors are usually isolated from the sWAT of young lean mice to obtain high differentiation efficiency. Here, we tested adipocyte progenitors isolated from the eWAT of older, HFD-fed mice, likely blunting any potential impact on adipogenesis by macrophages. Alternatively, PPARγ acetylation in macrophages may affect fibroblast expansion rather than adipogenic potential. Nevertheless, these data emphasize the diverse roles PPARγ acetylation could play, especially within inflammatory macrophages, in the pathological remodeling of adipose tissue.

In summary, using a conditional knock-in mouse model, our study provides evidence that acetylation of PPARγ in macrophages promotes infiltration into adipose tissue and impairs alternative activation, thus exacerbating visceral adipose tissue inflammation, fibrosis, and dysfunction. The absence of properly functioning macrophages propels adipose tissue degeneration, leading to metabolic derangement. Limitations of our data are the use of *LysM-cre* mice, which is not specific to ATMs. Therefore, macrophages in other tissues may also be affected by acetylation, which would necessitate other modeling. Mechanisms of PPARγ acetylation and deacetylation in macrophages also require further study. Collectively, our study highlights that a greater understanding of the function of PPARγ acetylation in macrophages will help in our understanding of adipose tissue responses to metabolic changes.

## Materials and methods

### Animal studies

The trinucleotide AAA coding for Lysine 293 (K293) in exon 6 of PPARγ was mutated to CAG to alter the coding of the residue to Glutamine (Q293) in linearized vectors (K293Q) and transfected into mouse embryonic stem cells for recombination. Antibiotic selection to generate G418-resistant clones was followed and assessed using Southern blotting and Long-Range PCR. Genomic DNA sequencing was completed for further confirmation. Chimeric mice were generated via blastocyst injection in mice with a C57Bl/6 background and mice carrying the K293Q allele were crossed with *LysM-cre* mice to produce myeloid-derived cell-specific mutations of K293Q. *K293Q*^*flox/flox*^*:LysM-cre* (mK293Q) homozygotes were used for all experiments and genotyped using PCR.

Mice were housed at room temperature (RT, 23 ± 1°C) on a 12 h light/12 h dark cycle with access to food and water *ad libitum*. The HFD contained 60% calories from fat, 20% from protein, and 20% from carbohydrates (Research Diets: D12492i). Rosi maleate (Avandia) (Abcam, ab142461) was mixed into the HFD at 100 mg/kg by Research Diets (New Brunswick, NJ) to achieve a dose of ~5 mg/kg BW. For the glucose tolerance test (GTT), mice were fasted overnight in cages with fresh bedding and injected intraperitoneally (i.p.) with glucose (2 g/kg BW). Blood glucose was measured with a One Touch Ultra glucometer at indicated time points. For the insulin tolerance test (ITT), mice were fasted for 4 h and injected i.p. with insulin (0.75 U insulin/kg BW). Body compositions were determined by EchoMRI. Plasma parameters were measured with HR Series NEFA-HR (Fujifilm catalog #9 9534791), Infinity Triglycerides Liquid Stable Reagent (Thermo Fisher Scientific catalog #TR22421), Mouse Insulin ELISA (Mercodia caralog #10–1247-01), and Mouse/Rat Leptin Quantikine ELISA kit (R&D Systems catalog #MOB00B). For calorimetric studies, we used the Comprehensive Lab Animal Monitoring System (CLAMS) (Columbus Instruments) with temperature-controlled settings.

### BMDM isolation

BMDMs were prepared from monocytes isolated from the tibia and femur of 6–8-week-old control and mK293Q mice as previously described [50]. Briefly, cells were incubated for 7 days in low-glucose Dulbecco’s modified Eagle’s medium (DMEM), 10% FBS, and 10 ng/mL macrophage colony-stimulating factor (Biolegend catalog #576404) to promote their differentiation into un-activated (M0) macrophages. Cells were then incubated in DMEM containing LPS (50 ng/mL) (Sigma catalog #L4524) to promote M1 activation or IL-4 (50 ng/mL) (Biolegend, catalog #574304) to promote M2 polarization.

### Adipose SVF isolation

Epidydimal fat pads were dissected control and mK293Q mice after 16 weeks on HFD, followed by mincing and digesting in Liberase (Sigma-Aldrich, catalog #5401127001) at 37°C for 30 min with gentle agitation. After passing through a 100 μm pore cell strainer, the SVF cells were pelleted by centrifuging at 400 × *g* for 5 min at 4°C.

### Hepatic lipid isolation

Liver lipids were isolated using the Folch Extraction Method. In brief, 50 mg of liver tissue was homogenized in PBS and mixed with a chloroform:methanol 2:1 solution. Samples were centrifuged and organic layers were collected, blown under nitrogen gas, and solubilized for immediate quantification using an Infinity Triglycerides Liquid Stable Reagent (Thermo Fisher Scientific catalog # TR22421).

### Quantitative real-time PCR (qPCR)

Tissue and cell RNA was isolated using the Tri-Isolate RNA Pure Kit (IBI Scientific IB47632). The High-Capacity cDNA Reverse Transcription Kit (Applied Biosystems) was used to synthesize cDNA from 1 μg total RNA. qPCR was performed on a Bio-Rad CFX96 Real-Time PCR system with the AzuraView Green Fast qPCR Blue Mix (Azura Genomics). Relative gene expression levels were calculated using the ΔΔCt method with CPA as the reference gene.

### Histological analyses

Tissues were collected and fixed in 10% neutral buffered formalin overnight at 4°C and kept in 70% ethanol at 4°C. Allocated sections were stained with H&E and imaged under a bright field microscope. Sections stained with Picrosirius red (Polysciences 24901) were imaged under both bright field and polarized light microscopy. Immunohistochemistry for F4/80 (CST, catalog #70076) was performed using a 1:100 dilution in PBST. The signal intensity was quantified by using ImageJ (NIH).

### Western blots

Cells were lysed and tissues were homogenized by Polytron homogenizer immediately after dissection in western extraction buffer (150 mmol/L NaCl, 10% glycerol, 1% NP-40, 1 mmol/L EDTA, 20 mmol/L NaF, 30 mmol/L sodium pyrophosphate, 0.5% sodium deoxycholate, 0.05% SDS, 25 mmol/L Tris-HCl: pH 7.4) containing protease inhibitor cocktail (Roche). The lysate was then sonicated, and debris was removed by centrifugation. SDS-PAGE and western blotting were performed and detected with ECL (Thermo Scientific). Antibodies used for western blot analysis were as follows: anti-Adipsin (R&D Systems, catalog #AF5430), anti-Adiponectin (Invitrogen, catalog # PA1–054), and anti-HSP90 (Proteintech,catalog #1371–1-AP).

### Luciferase reporter activity

Human embryonic kidney 293T (HEK293T) cells were cultured in DMEM containing 10% FBS (heat-inactivated) and 1% penicillin/streptomycin. Upon 50%−60% confluence, cells were seeded in 12-well plates for transfection using TransIT-LT1 (MirusBio # MIR2304) for 24 h, followed by 24-h treatment of 5 μmol/L Rosi. The following plasmids were used for transfection: pMCP-Luc (*Mcp-1* promoter in a pGL3-basic vector, Addgene #40324), pRL-SV40P (for normalization of luciferase experiments, Addgene #27163), pcDNA-Flag-HA-PPARγ2 WT, and K293Q mutant generated using site-directed mutagenesis, previously validated and tested [18, 19].

### Statistics

Values are presented as mean ± SEM. We used unpaired two-tailed Student’s *t*-test and two-way ANOVA to evaluate statistical significance with the assumption of equal variances. A *P* < 0.05 was considered to be statistically significant. Sample size estimation was decided based on past observations in similar studies, and details on sample size for mouse and cell culture experiments are included in each figure legend. Data were analyzed in GraphPad Prism (version 9.1.0).

## Supplementary Material

Supplementary Figures
