# Supplementary material for: Acetylation of PPARγ in macrophages promotes visceral fat degeneration in obesity
Source: Life Metab. 2022 Nov 11;1(3):258–69. doi: 10.1093/lifemeta/loac032 (PMC10198133; doi:10.1093/lifemeta/loac032)
Supplement: loac032_suppl_Supplementary_Figures [file loac032_suppl_Supplementary_Figures.docx]

**SUPPLEMENTARY INFORMATION**

**Acetylation of PPARγ in macrophages promotes visceral fat degeneration in obesity**

Nicole Aaron^1,2^*, Tarik Zahr^1,2^*, Ying He^1,3^, Lexiang Yu^1,3^, Brent Mayfield^1,4^, Utpal B. Pajvani^1,5^, Li Qiang^1,3^†

^1^Naomi Berrie Diabetes Center, ^2^Department of Pharmacology, ^3^Department of Pathology and Cell Biology, ^4^Department of Genetics and Development, ^5^Department of Medicine, Columbia University, New York, United States

* Contributed equally

† Correspondence: Li Qiang: [lq2123@cumc.columbia.edu](mailto:lq2123@cumc.columbia.edu)

**a b**

**Supplemental Figure 1:** Generation of the acetylation-mimetic K293Q knock-in mouse model.

(a) Schematic underlying the genetic approach to generate myeloid cell-specific mK293Q floxed mice. (b) qPCR analysis confirming the change in WT and mK293Q PPARγ gene expression in BMDMs of mutant mK293Q mice (n=4, 4). **p<0.01, ***p<0.001 for control group vs. mK293Q mice. Data represent mean ± SEM. Two-tailed Student’s *t*-test was used for statistical analysis.

**a b c**

**Supplemental Figure 2:** PPARγ acetylation does not impact mitochondrial activity of macrophages.

(a-c) qPCR analyses of mitochondrial metabolism-related gene expression from non-activated (M0) BMDMs (a), pro-inflammatory M1 activated macrophages (b), and anti-inflammatory M2 activated macrophages (c). Data represent mean ± SEM. Two-tailed Student’s *t*-tests were used for statistical analyses (n=3, 3).
